# Regulation of Adenine Nucleotide Metabolism by Adenylate Kinase Isozymes: Physiological Roles and Diseases

**DOI:** 10.3390/ijms24065561

**Published:** 2023-03-14

**Authors:** Koichi Fujisawa

**Affiliations:** Department of Environmental Oncology, Institute of Industrial Ecological Sciences, University of Occupational and Environmental Health, 1-1 Iseigaoka, Yahatanishi-ku, Kitakyushu 807-8555, Japan; fujisawa@yamaguchi-u.ac.jp; Tel.: +81-93-691-7469; Fax: +81-93-601-2199

**Keywords:** adenine nucleotides, adenylate kinase, energy metabolism, ATP, enzyme

## Abstract

Adenylate kinase (AK) regulates adenine nucleotide metabolism and catalyzes the ATP + AMP ⇌ 2ADP reaction in a wide range of organisms and bacteria. AKs regulate adenine nucleotide ratios in different intracellular compartments and maintain the homeostasis of the intracellular nucleotide metabolism necessary for growth, differentiation, and motility. To date, nine isozymes have been identified and their functions have been analyzed. Moreover, the dynamics of the intracellular energy metabolism, diseases caused by AK mutations, the relationship with carcinogenesis, and circadian rhythms have recently been reported. This article summarizes the current knowledge regarding the physiological roles of AK isozymes in different diseases. In particular, this review focused on the symptoms caused by mutated AK isozymes in humans and phenotypic changes arising from altered gene expression in animal models. The future analysis of intracellular, extracellular, and intercellular energy metabolism with a focus on AK will aid in a wide range of new therapeutic approaches for various diseases, including cancer, lifestyle-related diseases, and aging.

## 1. Introduction

Adenylate kinase (AK) is an enzyme that regulates adenine nucleotide metabolism which is widely present in higher organisms and bacteria, catalyzing the reaction ATP + AMP ⇌ 2ADP. AK is necessary for the normal functioning of organisms, including growth, differentiation, motility, and metabolism. Nine isozymes (AK1–AK9) have been identified and named according to their order of discovery [1,2]: AK1, AK5, AK7, and AK8 are localized in the cytoplasm [3,4,5]; AK2 in the mitochondrial intermembrane space; and AK3 and AK4 have been reported to be present in the mitochondrial matrix [6]. AK6 and AK9 are localized in the nucleus and regulate adenine nucleotide metabolism [7]. Although a lot of energy is required in the nucleus for various activities involved in gene expression, nuclear energy metabolism is not well understood. AK isozymes and CK isozymes localized in the nucleus, mitochondria, and cytoplasm, respectively, and mitochondrial translocation to the nuclear periphery are thought to be important for energy metabolism [8].

Organisms require energy for nucleic acid and protein synthesis and various biological reactions. The human body contains approximately 250 g of ATP, the main energy currency molecule, which is recycled and synthesized at an amount equal to the body weight per day [9]. Within the cell, ATP is produced in large quantities in the mitochondria; however, the efficient transfer of high-energy phosphate to intracellular sites where energy is required is an important aspect of intracellular energy metabolism. In addition to the simple diffusion of ATP, creatine kinase (CK), AK, mitochondrial migration, and extracellular vesicles (EVs) in the system, including the extracellular region, are thought to support the energy network when high-energy phosphate is transferred from the mitochondria to places where energy is required. ATP diffusion has been studied intensively using highly energy-demanding cells such as muscle cells [10].

EVs are a generic term for various types of membrane components ranging from 20 to 1000 nm in diameter released by numerous cell types. Exosomes, a type of EVs, carry various biomolecules, such as proteins, nucleic acids, and lipids. Exosomes mediate short- and long-distance communication between cells [11]. Exosomes have attracted a great deal of attention in the field of cancer research because they have been shown to promote the malignant transformation of cancer and acquisition of drug resistance [12]. Additionally, exosomes secreted by oxaliplatin-resistant colon cancer cells enhance the glycolytic system of non-resistant cancer cells, induce increased glucose uptake, and promote lactate and ATP production, causing the cells to mutate into drug-resistant cancer cells [13]. Tumor-derived exosomes can support metastatic progression in some tumors by interacting with the microenvironment and can serve as tumor biomarkers. Exosomes induce various changes in cellular functions via their own internalized transmitters; therefore, an in-depth analysis of the functions of AK in EVs is important. Exosomes with AK may be useful not only for cancer treatment but also for regenerative medicine. Moreover, mesenchymal stem cell (MSC) exosomes contain glycolytic enzymes, namely nucleoside diphosphate kinase and adenylate kinase [14]. In addition, MSC-derived exosomes promote myocardial viability and cardiac function by activating the PI3K/Akt pathway, increasing ATP levels, and reducing oxidative stress in cells with myocardial ischemia/reperfusion injury [15].

**Figure 1:**
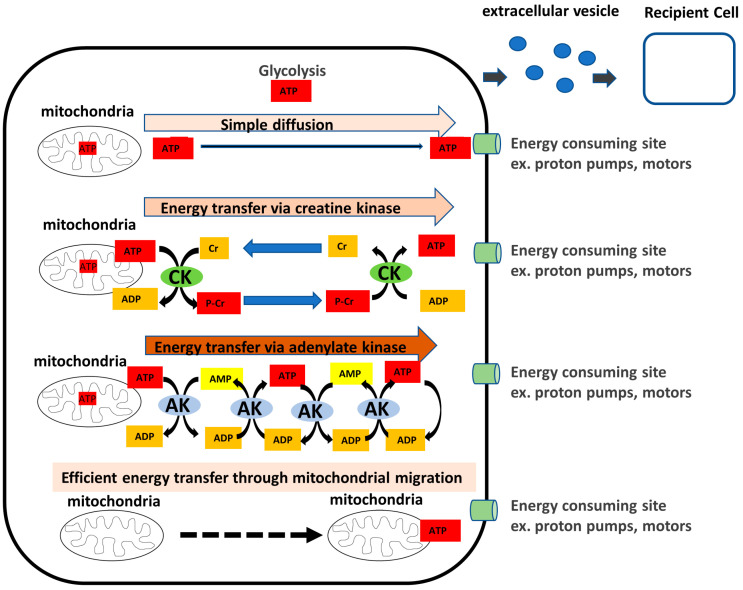
Model for high-energy phosphoryl transfer via adenylate kinase. As energy is transported from the mitochondria to the sites of utilization such as proton pumps or motors, the intracellular system supports an energy network of creatine kinase (CK), adenylate kinase (AK), and the localization or transport of the mitochondria themselves, in addition to simple diffusion. The presence of extracellular secretory AKs outside the cells suggests the existence of extracellular energy metabolism. In addition, intercellular energy networks, such as extracellular vesicles (EVs) are thought to exist.

AK has been proposed as an efficient model for high-energy phosphate transfer (Figure 1). However, the existence of extra- and intercellular AK suggests that there may be an unknown mechanism of energy metabolism, not only within the cell but also outside the cell and in the intercellular space. This article summarizes our current understanding of the physiological roles of AK isozymes and associated diseases.

## 2. Characteristics and Diseases Related to the Adenylate Kinase Isozymes

We summarize the phenotypes of the associated diseases and expression changes of each AK isozyme listed in Table 1 and the AK isozymes contained in exosomes obtained from vesiclepedia databases (http://microvesicles.org (accessed on 17 February 2023)) in Table 2.

### 2.1. AK1

#### 2.1.1. AK1 Exists in the Cytoplasm and in the Extracellular Space

AK1 is a cytosolic enzyme with the highest expression levels in the skeletal muscle, brain, and erythrocytes [1]. ATP is released not only into the cell, but also out of the cell, where it acts as an information transduction molecule. The ability of human and mouse sera to transphosphorylate ATP and AMP into ADP suggests the presence of soluble forms of AK [16,17]. A major soluble AK isoform, AK1, has been shown to circulate freely in the blood and to control the majority of ADP homeostasis in cell-free serum [18]. Furthermore, AK1 was identified as one of the proteins common to both apical secretions, from well-differentiated primary human tracheobronchial cells grown at the air–liquid interface, and human tracheobronchial normal induced sputum [19]. AK1 is also important as a signal transduction mechanism, and future research on the function of AK1 outside the cell is expected.

#### 2.1.2. AK1 Deficiency Causes Hematological Abnormalities

AK1 deficiency has been reported to cause hemolytic anemia. Chronic hemolytic anemia has been reported in an Arab sister and brother with low AK activity in red blood cells [20], a French girl with low AK activity in red blood cells which was mentally disabled [21], and a heterozygous Japanese girl with AK activity reduced by approximately 50% [22]. Hemolytic anemia associated with complete AK1 deficiency has also been reported in an American girl [23] and that associated with GTP:AMP phosphotransferase deficiency in a Syrian girl [24]. The genetic analysis of a case reported in Japan showed an Arg to Trp substitution [25]. Additionally, moderate to severe chronic non-spherocytic erythrocytic hemolytic anemia due to decreased AK1 activity caused by gene mutations, including missense, nonsense, frameshift, deletion, and substitution mutations, has been reported [23]. Nonsense mutations in two siblings with the most severe erythrocyte AK deficiency and mental disabilities was attributed to the complete absence of AK [26]. Cases of mental retardation associated with hemolytic anemia have also been reported [27,28]. Recently, another case of moderate erythrocyte AK1 deficiency associated with chronic non-spherocytic hemolytic anemia in China was reported, suggesting that decreased AK1 protein enzyme activity may cause AMP-activated protein kinase (AMPK) dysregulation, which in turn leads to glycogen synthase kinase-3 beta activation and neurodegeneration [29].

#### 2.1.3. AK1 Expression Decreases in Non-Obstructive Azoospermia Patients

A proteomic study of testicular biopsies from non-obstructive and obstructive azoospermia patients screened for genes involved in spermatogenesis defects. AK1 was found to be expressed in non-obstructive azoospermia. The AK1 expression was significantly decreased in patients with non-obstructive azoospermia than in patients with obstructive azoospermia. Furthermore, testicular biopsy tissue analysis showed that AK1 protein levels were significantly decreased in patients with non-obstructive azoospermia, suggesting a link between decreased AK1 protein levels and defects in spermatogenesis. AK1 destruction greatly impairs sperm motility under energy stress conditions where only ADP is available [30]. Sperms require flagellar motility until they encounter the oocyte, thereby utilizing ATP, suggesting that energy metabolism is important in this process. The relationship between AK and male infertility has attracted attention because, as described below, AK7 has been shown to be involved in abnormal spermatogenesis, and AK8 involvement has been reported in sperm motility.

#### 2.1.4. AK1 Expression Levels Are Associated with Behavioral Activity in an Animal Model

Spontaneously Running-Tokushima-Shikoku (SPORTS) rats are a hyperactive rat strain. However, the causative mutation for this phenotype has not yet been identified. Among adenylate kinase isozymes that maintain the homeostasis of a cellular adenine nucleotide composition in the cell, only AK1 was highly upregulated in both exercised and sedentary SPORTS rats compared to that in wild-type (WT) rats [31].

Medaka fish overexpressing AK1 also show increased spontaneous locomotor activity compared to that in the WT. Interestingly, this increase is temperature-dependent, suggesting that cellular energy balance may regulate spontaneous locomotor activity [32]. The importance of AK1 at the behavioral level should be analyzed in the future.

#### 2.1.5. AK1 Knockout Affects Muscles, Heart Muscle, and Sperms

AK1 knockout mice were the first model to be reported among AK isozymes. The genetic disruption of AK1 dysregulates the muscle energetic economy and decreases tolerance to metabolic stress, despite rearrangements in alternative high-energy phosphoryl transfer pathways such as glycolytic, guanylate, and creatine kinase phosphotransfer pathways [33]. Furthermore, AK1 deficiency has been reported to induce fiber-type specific variation in groups of transcripts involved in glycolysis and mitochondrial metabolism, as well as in gene products that define structural and myogenic events [34]. Moreover, during the onset of ischemia, AK1 knockout mouse hearts exhibit an accelerated loss of contractility and reduced tolerance to ischemic stress compared to that in WT controls [35].

#### 2.1.6. AK Isozymes and Other Enzymes Complement Each Other under AK1 Deficiency

The simultaneous disruption of cytoplasmic muscle type-CK and AK1 isozyme genes has been shown to impair intracellular energy communication and greatly reduce the cellular ability to maintain the total ATP metabolic turnover during muscle function [34]. Thus, under normal conditions, AK isozymes and other isozymes complement each other; however, under metabolic stress, complementation becomes insufficient and symptoms are observed. Further analysis of the AK isozyme network will be important in understanding its role in cellular homeostasis and functions.

### 2.2. AK2

#### 2.2.1. AK2 Regulates Energy Metabolism in the Mitochondrial Intermembrane Space

AK2 is specific to the mitochondrial intermembrane region and is highly expressed in the liver, kidneys, and heart [36,37]. It is suggested to be involved in supplying ADP to the adenine nucleotide transporter (ANT) localized in the mitochondrial inner membrane [38]. AK2 functions as an essential regulator of mitochondrial apoptosis through AK2-Fas, associated via death domain (FADD)-caspase 10 [39]. However, the exact role of AK2 in the mitochondrial intermembrane space remains unknown.

#### 2.2.2. AK2 Deficiency Causes Reticular Dysgenesis

AK2 deficiency has been reported to be the cause of reticular dysgenesis (MIM 267500), an acute form of severe combined immunodeficiency, which is characterized by the concomitant occurrence of congenital agranulocytosis and bilateral sensorineural deafness [40]. One missense mutation, two small and one large deletion and two splice site mutations have been reported toward human immunodeficiency syndromes that cause reticular dysgenesis and can be classified as mitochondrial [41]. Additionally, the first non-normal splice site pathogenic mutation in AK2 led to reticular dysgenesis in the homozygous state [42]. AK2 function has been evaluated in vitro, where it is significantly induced during adipocyte and B-cell differentiation. Its depletion by RNAi is associated with adiponectin secretion in 3T3-L1 adipocytes and BCL1 cells, IgM secretion sein BCL1 cells, and impaired unfolded protein response (UPR) induction during the differentiation of both cell types. Furthermore, the etiology of severe hematopoietic defects in reticular dysgenesis, a disease associated with AK2 gene mutations in humans, may be attributed to the requirement of AK2 for UPR induction [43]. AK2-deficient myeloid progenitors are also unable to effectively use ATP in their mitochondria, resulting in defects in ATP transport from the mitochondria to the endoplasmic reticulum during neutrophil differentiation. Thus, neutrophil differentiation is affected, while macrophage differentiation can be maintained by activating transcription factor (ATF) 6 activation in the absence of mitochondrial ATP supply because ATF6 activation does not require ATP [44].

#### 2.2.3. AK2 Knockout in Animals Is Lethal

In unicellular organisms, such as *Escherichia coli* and *Schizosaccharomyces pombe*, which have only one prototype of AK2, the disruption of the AK gene is lethal [45]. AK2 knockout in Drosophila is reported to be lethal at the larval stage [1]. Transgenic AK2^−/−^ null homozygosity was reported to be lethal in the early embryo, indicating that AK2 is essential for in utero development. In adults, the conditional organ-specific ablation of AK2 caused abrupt heart failure with an accumulation of the Krebs cycle and glycolytic metabolites, suggesting a vital contribution towards a highly energy-demanding cardiac performance. Interestingly, a compensatory upregulation of phosphotransferases AK1, AK3, AK4 isozymes, CK isoforms, and hexokinase, along with the remodeling of cell cycle/growth genes and mitochondrial ultrastructure, supported organ rescue [46]. Furthermore, aberrant leukocyte development occurs in zebrafish upon AK2 knockdown [41]. AK2 deficiency is also known to affect hematopoietic stem and progenitor cell development, increasing oxidative stress and apoptosis. Zebrafish models have also shown that AK2 is required for the appropriate development, survival, and regeneration of sensory hair cells. Interestingly, AK2 deficiency induces the expression of several oxidative stress markers and causes increased death in hair cells [47]. Furthermore, Rissone et al. have reported on the roles that AK2 plays in the development, survival, and regeneration of sensory hair cells using AK2-deficient zebrafish models [48]. AK2 is the only AK isozyme that localizes to the mitochondrial intermembrane space and may play an important role in energy transfer from mitochondria to the cytoplasm and nucleus. It is expected that various types of conditional knockout mice will aid in elucidating the roles of AK2 in different organs.

#### 2.2.4. AK2 Expression Shows Circadian Variation

Nucleotides are primarily regulated in the liver by salvage or de novo synthesis, and hepatic nucleotide metabolism is regulated by the diurnal rhythmic variation of metabolites from the transcriptional control of rate-limiting genes. Loss of the local hepatic circadian clock is believed to cause significant perturbations in the normal rhythm of nucleotides, affecting physiological functions through the AMPK pathway, whose activity is allosterically regulated by the AMP/ATP ratio [49]. Due to the circadian abundance of ATP, much attention has been paid to the metabolism–clock relationship [50,51,52]. AK2 knockout Drosophila studies have suggested its role in the generation of circadian rhythms [1]. Additionally, the rhythmic expression of mitochondria-localized AK2 and AK4 has been shown to occur using protein variation data from the liver [53].

#### 2.2.5. AK2 Suppresses Cancer

AK2 mediates mitochondrial apoptosis and functions in concert with FADD and caspase-10, indicating its role in a novel intrinsic apoptotic pathway involved in tumorigenesis [39]. Additionally, AK2 is an activator of the dual-specificity protein phosphatase 26 and suppresses cell proliferation through the dephosphorylation of FADD, suggesting that AK2 is a negative regulator of tumor growth. Furthermore, AK2 is an AMP-sensing negative regulator of BRAF (B-Raf proto-oncogene) in tumorigenesis, and monitors cellular AMP levels linking metabolic status to the tumor [54]. AK2 has also been shown to play a tumor-suppressive role; a low expression of AK2 correlates with poor prognosis in patients with HCC.

#### 2.2.6. AK2 Promotes Cancer

The higher expression of AK2 is associated with worse prognosis. AK2 is overexpressed in lung adenocarcinoma, and the positive expression of AK2 is associated with tumor progression and reduced survival in patients. The knockdown of AK2 inhibits the proliferation, migration, and invasion of human lung adenocarcinoma cells, induces apoptosis and autophagy. AK2 knockdown cells have been shown to exert greater tumor suppression in vitro and in xenograft mice [55]. Similarly, the knockdown or knockout of AK2 inhibits the migration and invasive potential of human lung adenocarcinoma cells, while its overexpression promotes cancer metastasis, providing a rationale for targeting AK2 as a therapeutic approach for lung cancer [56]. Furthermore, the analysis of exosomes involved in neuroblastoma metastasis revealed that AK2 were upregulated in bone-marrow-metastasis-derived exosomes compared to primary tumor-derived exosomes [57]. The paradox of whether AK2 acts in a cancer-suppressive or cancer-promoting manner has been suggested to differ depending on the cancer cell type and developmental stage [58] and needs to be assessed further.

### 2.3. AK3

#### 2.3.1. AK3 Regulates Energy Metabolism in the Mitochondrial Matrix

The AK isozyme AK3, constitutively expressed in a variety of tissues, is present in the mitochondrial matrix together with AK4 and may be closely involved in mitochondrial energy metabolism. AK3 is believed to reversibly mediate the Mg^2+^ GTP + AMP ↔ Mg^2+^ GDP + ADP reaction [1]. It is reported that AK3 renders cisplatin-resistant cancer cells susceptible to tobacco condensate vapor exposure [59]. Although AK3 expression is low in pancreatic beta cells because they do not consume GTP, which is required for insulin secretion [60], its definite function has not been clarified. Recently, AK3 knockout in HeLa cells has been reported to decrease intracellular ATP levels; however, knockout mice studies are lacking and must be the direction of future research [61].

#### 2.3.2. AK3 Expression Level Is Associated with Survival in Cancer Patients

The Cancer Genome Atlas (TCGA) for the detection of a new genetic signature to predict breast cancer prognosis has shown that AK3 is one of the seven genes that correlates with the glycolytic system, and its higher expression is associated with a better prognosis. In bladder urothelial carcinoma, the decreased expression of AK3 was associated with a worse prognosis, and in breast cancer, decreased AK3 levels were significantly associated with decreased overall survival [62]. Therefore, AK3 is considered a useful marker [63].

### 2.4. AK4

#### 2.4.1. AK4 Is Involved in Oxidative Stress

AK4, which is localized in the mitochondrial matrix along with AK3, has been suggested to be involved in oxidative stress. AK4 is one of the proteins that are upregulated by the administration of four hepatotoxic drugs including carbon tetrachloride [64]. AK4 interacts with adenine nucleotide translocase as a stress-responsive protein to maintain cell survival [65]. Additionally, AK4 is one of the genes regulated dependently by NAD (P)H oxidase, an enzyme involved in the regulation of reactive oxygen species [66].

#### 2.4.2. AK4 Regulates Mitochondrial Activity under Hypoxia

The intracellular and tissue-specific expression of AK4 has been extensively studied [6]. Interestingly, an siRNA screen of 2000 genes localized in mitochondria reported that AK4 is the most important gene involved in ATP production in the mitochondria [67]. It has been speculated to be associated with the suppression of mitochondrial activity by hypoxia, since it is markedly induced by hypoxia inducible factor 1α (HIF-1α). Chronic hypoxia has recently been reported to upregulate the AK4 expression in pulmonary arterial smooth muscle cells in a HIF-1α-dependent manner. AK4 knockdown reduces the viability and proliferation of pulmonary arterial smooth muscle cells under both normoxia and chronic hypoxia. AK4 silencing in pulmonary arterial smooth muscle cells has enhanced the mitochondrial respiration and decreased the glycolytic metabolism [68]. However, AK4, along with its role in mitochondrial regulation, is yet to be studied in detail.

#### 2.4.3. AK4 Is Involved in Drug Resistance of Cancer Cells

AK4 expression in HeLa cells and hepatocellular carcinoma cells is induced by the administration of iron chelators, which mimic hypoxia and suggest a link to mitochondrial energy metabolism [69]. The knockdown of AK4 in cancer cells and further gene expression and metabolomic analyses revealed that it is important for the regulation of mitochondrial activity and that AK4 knockdown increases cancer drug sensitivity [70]. Its involvement in drug resistance has also been reported. Chronic exposure to tamoxifen induces an increase in m^6^A in the 5′ UTR of AK4 mRNA and promotes its translation, with high levels of AK4 inhibiting mitochondrial apoptosis and promoting ROS production, thereby activating p38 and ultimately MCF-7 in tamoxifen cells, which has been reported to increase the cell tolerance to tamoxifen [71].

#### 2.4.4. AK4 Is a Useful Prognostic Marker for Cancer

A relationship between AK4 and cancer has been reported, and it is suggested to be a carcinogenic or therapeutic target. Increased AK4 expression is observed in patients with bladder cancer and is associated with poor prognosis, while its inhibition in bladder cancer cell lines is associated with the suppression of cancer cell growth and decreased metastasis. These indicate that AK4 may have high potential as a therapeutic target [72]. AK4 is upregulated in lung adenocarcinomas compared to that in normal cells, and high AK4 expression has been reported to be associated with advanced stage, disease recurrence, and poor prognosis. The loss of AK4 expression led to the suppression of the invasive potential of lung cancer cell lines, whereas AK4 overexpression promoted the invasion in vitro and in vivo. Therefore, AK4 promotes malignant progression and recurrence by promoting metastasis in an ATF3-dependent manner [73]. AK4 overexpression promotes lung cancer metastasis by enhancing HIF-1α stability and epithelial–mesenchymal transition (EMT) under hypoxic conditions. Withaferin-A suppresses the AK4-HIF-1α signaling axis and serves as a potent anti-metastatic agent against lung cancer, suggesting that it may be a novel therapeutic agent [74]. AK4 localization to the mitochondria and induction of its expression by hypoxia suggests that AK4 is involved in the malignant transformation and metastasis of cancer. However, the detailed mechanism of mitochondrial regulation of AK4 is not known, and it will be important to analyze its function.

#### 2.4.5. AK4 Pseudogene Is Abundant in Circulating Exosomes in Patients with Pancreatic Adenocarcinoma

Notably, AK4P1, a pseudogene of AK4, was recently identified as an exosomal long non-coding (lnc) RNA whose abundance in circulating exosomes from pancreatic adenocarcinoma patients’ lncRNA is considerably higher than in circulating exosomes from healthy individuals [75]. The function and mechanisms associated with this gene are still unknown, and future analysis is expected.

### 2.5. AK5

#### 2.5.1. Anti-AK5 Antibodies Are Associated with Non-Paraneoplastic Limbic Encephalitis

Of the nine members of the AK family, only AK5 is known to be mainly expressed in the brain, primarily located in the cytoplasmic fraction [76]. AK5 is also expressed in other tissues, such as the intestine, although at a lower level than that in the brain. Limbic encephalitis with antibodies against AK5 is a non-paraneoplastic autoimmune disease that has been reported in a few patients. Its clinical presentation is characterized by severe amnesia, mood disturbance, and, interestingly, the absence of seizures in most cases [77]. Although the antibodies to AK5 may have been induced after neurological damage due to encephalitis of unknown etiology, the detailed cause remains unknown.

#### 2.5.2. AK5 Suppresses Cancer and Cell Growth through the AMPK/mTOR Pathway

The aberrant methylation of the promoter region of AK5 has been reported in a study investigating the aberrant methylation of CpG islands in human breast cancer [78]. AK5 is also significantly hypermethylated in colorectal cancer. It regulates AMPK and mTOR phosphorylation and inhibits cell migration and invasion in colorectal cancer cell lines. AK5 expression is suggested to regulate AMPK/mTOR signaling and may be closely associated with the metastasis of colorectal adenocarcinoma [79]. Its expression in colon adenocarcinoma tumor tissue is lower than that in noncancerous tissue; particularly, patients with high AK5 expression have longer overall survival than those with low expression. Furthermore, patients with low AK5 expression promoted cell proliferation and metastasis by regulating cell cycle pathways. Notably, in vivo results indicate that a reduced AK5 expression is required for tumor growth. However, in gastric cancer, patients with high AK5 levels in tumor tissue have significantly shorter survival rates compared to that in the low expression group. Moreover, the AK5 expression is associated with cancer stages and types and is an independent prognostic factor [80]. Its knockdown in AZ521 and MKN74 cells significantly inhibited growth and autophagy and increased apoptosis.

### 2.6. AK6

#### 2.6.1. AK6 Is Involved in Energy Metabolism in the Nucleus

AK6, also known as the coiled-interacting nuclear ATPase protein (CINAP), is primarily localized in the nucleus and has been characterized by crystal structure determination and enzyme assays. Unlike other AKs, human CINAP (hCINAP) has been shown to function as both an AK and an ATPase [7]. It rapidly generates ATP during intense activity, and this mechanism of action is considered to be a reserve energy system that can regenerate ATP from ADP under conditions of energy stress. AK6 is thought to be involved in nucleotide metabolism and energy communication between the mitochondria and nuclei via a phosphorylation transfer network. Human AK6 interacts with collin, a marker protein of Cajal bodies that is known to be involved in nuclear splicing [81]. The adrenal gland protein, AD-004-like protein, of *Caenorhabditis elegans* was a functionally unknown protein containing 182 amino acids. It was considered to be AK6 based on the measurement of enzyme activity and its localization to the nucleus when expressed in HeLa cells. The RNAi knockdown of AK6 led to a slow growth defect resulting from disrupted RNA splicing in the nucleus [82,83]. Fap7, the yeast hCINAP homologue, is shown to interact with the small subunit ribosomal protein Rps14, assembling a protein–RNA complex, and is essential for 18S rRNA maturation [24,84]. AK6 has also been identified in Drosophila, and this novel adenylate kinase is named as DAK6 [85].

#### 2.6.2. AK6 Is Associated with Poor Cancer Prognosis

AK6, upregulated under hypoxic conditions, is shown to be involved in metastasis, with HIF-1α and aryl hydrocarbon receptor nuclear translocator (ARNT) recruited to the promoter of AK6. Furthermore, the suppression of AK6 expression has been reported to reduce the migratory ability and EMT of cervical cancer cells under hypoxic conditions, indicating that it may be a potential therapeutic target for cervical cancer [86]. The loss of AK6 in fast-growing human cancer cells inhibits ribosome assembly and abolishes tumorigenesis. In humans, AK6 is known to be highly expressed in cancer and correlates with poor prognosis, and genome-wide polysome profiling has shown that hCINAP selectively regulates cancer-associated translatomes and promotes malignant transformation, making it a potential target for cancer therapy [82]. Interestingly, AK6 positively regulates lactate dehydrogenase A activity in cancer stem cells, producing more extracellular lactate, providing a favorable microenvironment for the growth and invasion of colorectal cancer stem cells, as well as promoting metabolic changes, thereby suppressing cancer stem cell ROS overproduction and promoting survival [87,88].

### 2.7. AK7

#### 2.7.1. AK7 Deficiency Causes Primary Ciliary Dyskinesia and Infertility

AK7 is expressed in ciliated tissues and testes, is associated with ciliary function, and is the only AK that contains a C-terminal Dpy-30 domain. The absence of AK7 orthologs from organisms lacking motile (9 + 2) cilia, such as *D. melanogaster* and *C. elegans*, in the plant and fungal kingdoms suggests the evolutionary conservation of this gene as an integral component of (9 + 2) microtubular organization [89]. Ultrastructural and functional defects in axonemes are known to cause primary ciliary dyskinesia, a disorder characterized by recurrent airway infections, epiphora, male sterility, and in the most severe cases, hydrocephalus [90]. Two mutations have been identified in AK7 in humans: a single nucleotide polymorphism (rs2369679) and (c.1214 insT). Family and functional studies have shown that c.1214 insT is associated with primary ciliary dyskinesia [91]. A genetic study of two siblings presenting with morphological anomalies of the flagella without features of respiratory primary ciliary dyskinesia also reported a c.2018T > G (p.Leu673Pro) mutation in AK7 [92]. Recently, a homozygous missense mutation in AK7 identified male infertility due to the multiple morphological anomalies of the flagella, and whole-genome sequencing showed two consanguineously related individuals with flagellar polymorphism abnormalities and oligoasthenoteratozoospermia [93]. Although the actual function of AK7 is unclear, it is assumed to participate in energy-relay mechanisms along the length of the axoneme through ATP regeneration.

#### 2.7.2. AK7 Knockout Mice Exhibit Primary Ciliary Dyskinesia

AK7 deficient mice exhibited pathological signs that are characteristic of primary ciliary dyskinesia, such as ultrastructural ciliary defects and reduced frequency of ciliary beating in the respiratory epithelium. It has reported to be associated with hydrocephalus, abnormal spermatogenesis, mucus accumulation in the sinuses, and dramatic respiratory pathology during allergen challenge. Furthermore, AK7-deficient male mice showed impaired spermatogenesis, that a large number of abnormal spermatozoa were observed in the seminiferous tubules, and that the axon structure was disorganized in the spermatozoa of Ak7-deficient male mice [3].

#### 2.7.3. AK7 Is A Useful Prognostic Marker for Ovarian Cancer

A study comparing the AK7 expression in normal and ovarian cancer tissues in the TCGA database and evaluating the correlation between AK7 levels and clinical manifestations of ovarian cancer showed that the AK7 levels were significantly downregulated in cancer tissues compared to that in normal ovarian tissues. Lower AK7 levels are associated with the patient age. Furthermore, the median overall survival of patients with low AK7-expressing ovarian cancer was shorter than that of patients with high AK7-expressing ovarian cancer, indicating that it may be a prognostic marker for ovarian cancer [94].

### 2.8. AK8

#### 2.8.1. AK8 Is Associated with the Axoneme

Cytoplasmic AK8, like AK5, has been reported to have two complete and active AK domains within its polypeptide chain [2]. AK8 is reported to be associated with the axoneme and may allow for the buffering of ATP among the axoneme and other compartments of the flagellum [95]. However, no disease associated with AK8 has been reported in humans.

#### 2.8.2. AK8 Knockout Causes Hydrocephalus

All AK8 knockout mice develop mild-to-moderate hydrocephalus, usually confined to the lateral ventricles. Despite the development of hydrocephalus, mutant mice are functional and exhibit normal behavior and growth. Time course studies have shown that hydrocephalus is not seen in embryos or neonates but becomes apparent at approximately 2 weeks of age and reaches maximum severity at weaning [96].

### 2.9. AK9

Previously known as *C6orf199*, a novel gene with an uncharacterized function was characterized as AK9 because of its sequential similarity and AK activity [97]. It is present in the cytosol and nucleus, with high expression levels in the pituitary gland, trachea, thymus, testis, and mammary gland, and moderate expression in the brain, pharynx, uterus, spleen, and lymph nodes. AK9 has been identified as a novel disease modifier in congenital myasthenic syndrome by altering nucleotide sugars in the N-glycosylation pathway [98]. Currently, no phonotype of transgenic or knockout mice has been reported and awaits further evaluation.

**Table 1:** Phenotypes of associated diseases and expression changes in each AK isozyme.

Isozyme	Intracellular Localization/ Highly Expressed Tissues	Physiological Characteristics	Related Human Diseases	Animal Model/Cultured Cell
AK1	Cytosol/nuscle, brain, heart, testis	Highly expressed in skeletal muscles, brain, and erythrocytes [1]	Non-obstructive azoospermia [30] Hematological abnormality [20,21,22]	KO mouse, inhibited sperm motility under specific conditions [30,33,99] Tg medaka, increased spontaneous locomotor activity [32] and reduced tolerance to ischemic stress [35]
AK2	Mitochondrial intermembrane space/liver, kidney, heart	Supply of ADP to the adenine nucleotide transporter [100] Regulation of mitochondrial apoptosis [39] Transfer of energy from the mitochondria [36]	Reticular dysgenesis [40,41,42,47]	KO drosophila, lethal at the larval stage [1] KO zebrafish, reticular dysgenesis, and affected sensory hair cells [48] KO mouse, lethal in the early embryo [46]
AK3	Mitochondrial matrix/all tissues except for red blood cell	Mitochondrial energy metabolism [1] Low expression in pancreatic beta cells [60]	Not reported	HeLa cell, slow growth [61]
AK4	Mitochondrial matrix/kidney, liver, brain, heart, stomach, intestine, gonads	Mitochondrial energy metabolism [6,67] Hypoxia-related mitochondrial regulation [67,69,70]	Not reported	Smooth muscle cells, AK4 KD enhanced mitochondrial respiration and decreased glycolytic metabolism [68] Hela cell, AK4 KD decreased mitochondrial activity [70]
AK5	Cytosol nucleus/highly expressed in brain	Energy regulation in brain [76]	Non-paraneoplastic limbic encephalitis [77]	AZ521 and MKN74 cells, AK5 KD inhibited growth and autophagy and increased apoptosis [80]
AK6	Nucleus/all tissues	Nuclear energy metabolism [81]	Not reported	*C. elegans*, growth inhibition [82,83]
AK7	Cytosol/lung, trachea, testis, mammary gland tissue	Expressed in ciliated tissues and testes [3]	Respiratory primary ciliary dyskinesia [91] Morphological anomalies of the flagella [92] Multiple morphological anomalies of the flagella, Oligoasthenoteratozoospermia [93]	KO mouse, primary ciliary dyskinesia hydrocephalus, abnormal spermatogenesis, mucus accumulation in the sinuses, and impaired spermatogenesis [3]
AK8	Cytosol/liver, paancreas, lung, testis	Associated with the axoneme [95]	Not reported	KO mouse, hydrocephalus [96]
AK9	Cytosol, nucleus/ highly expressed in pituitary gland, trachea, thymus, testis, mammary gland	Highly expressed in the pituitary gland, trachea, thymus, and testis [97]	Congenital myasthenic syndrome [98]	Not reported

KO: knockout, KD: knockdown, Tg: transgenic.

**Table 2:** AK isozymes in exosomes.

Isozyme/Entrez Gene ID	Tissue/Cell type	Reference
AK1/203	Ovarian cancer cells	[101]
AK1/203	Platelets	[102]
AK1/203	Squamous carcinoma cells	[103]
AK1/203	Colorectal cancer cells	[104]
AK1/203	Colorectal cancer cells	[105]
AK1/203	Urine	[106]
AK1/203	Urine	[107]
AK2/204	B-cells	[108]
AK2/204	Colorectal cancer cells	[104]
AK2/204	Colorectal cancer cells	[105]
AK2/204	Dendritic cells	[109]
AK2/204	Endothelial cells	[110]
AK2/204	Ovarian cancer cells	[101]
AK2/204	Prostate cancer cells	[111]
AK2/204	Squamous carcinoma cells	[103]
AK3/50808	Hepatocytes	[112]
AK4/205	Squamous carcinoma cells	[103]
AK4/205	Urine	[113]
AK4 pseudogene 3/645619	Glioblastoma cells	[114]
AK5/26289	Glioblastoma cells	[114]
AK5/26289	Mesenchymal stem cells	[115]

## 3. Conclusions

Energy metabolism is involved in many diseases, including lifestyle-related diseases, such as diabetes and fatty liver, aging, cancer, and neurodegeneration, and has been particularly studied in recent years. Although AK has been studied quite extensively, recent findings have shown its interesting relation to the dynamics of cellular energy metabolism, cancer, and circadian rhythm. AMPK is an intracellular AMP sensor that senses a decrease in AMP and becomes activated under conditions of energy deficiency. AK is closely related to AMPK and is considered to be a metabolic monitor. Since the energy metabolism is also closely related to fatty acid oxidation, autophagy, and aging, the analysis of intracellular energy metabolism will lead to a new understanding of many diseases. In particular, AK has been implicated in migration capacity, hypoxia, and drug resistance related to multi-organ metastasis in cancer research and is attracting attention as a new therapeutic target and prognostic marker for cancer. Analysis of extracellular AK is expected to be useful for regenerative medicine. This review summarizes current knowledge on the physiological roles of AK isozymes and related diseases; however, further analysis of the details of intracellular, extracellular, and intercellular energy metabolism, especially AK, will lead to new therapeutic strategies for various diseases, such as carcinogenesis, lifestyle-related diseases, and aging.

## Data Availability

Not applicable.
